# The Potential of 13C Isotopomers as a Test for the Vibrational Theory of Olfactory Sense Recognition

**DOI:** 10.1155/2013/515810

**Published:** 2013-05-26

**Authors:** Karel D. Klika

**Affiliations:** Molecular Structure Analysis, German Cancer Research Center (DKFZ), Im Neuenheimer Feld 280, D-69009 Heidelberg, Germany

## Abstract

The continuing debate over the basis of odorant recognition with respect to the molecular shape (“lock and key”) theory versus the vibrational theory could potentially be resolved by the testing of ^13^C-labeled odorants. The application of ^13^C isotopomers is discussed herein by means of DFT-calculated IR vibrations and Gibbs' free energies (Δ*G*) for acetophenone and octan-1-ol, two odorants for which the ^2^D (deuterium) isotopomers have previously been shown to be discernible from their respective ^1^H (normal) counterparts by *Drosophila melanogaster*.

## 1. Introduction

For many animals, their ability to detect volatile molecules-odorants-by way of the olfactory sense can literally mean the difference between survival and demise or the continued propagation of the species. By contrast, for human beings, our sense of smell, which is weak in comparison to some animals, is considered more of an indulgence rather than a functionality necessary for survival on a par with sight and hearing, perhaps thereby explaining in part, from an anthropocentric perspective, why it is the least understood of our senses. Although olfactory receptor (OR) sites have been identified and the molecular basis for the olfactory sensory process elucidated (both in vertebrates and insects for which the processes differ [1])—indeed, the work warranted the awarding of a Nobel Prize to Buck and Axel [2–4] for this outstanding accomplishment—the means by which odorants are recognized at the molecular level and actuation occurs is still debated. Thus, intriguingly, despite numerous attempts and decades of endeavor, there is still no definitive consensus on the identity of the molecular property that the OR is responding to, and the manner of odorant recognition remains, surprisingly, a contentious issue [5–15]. Overall, there are, or have been over the years, a number of competing theories, either distinct or modified, and these theories or variations thereof number in the dozens [6, 15, 16]. It is, however, generally accepted [1, 6, 15, 17] presently by mainstream researchers that the molecular basis for odorant recognition by an OR is essentially based on the molecular size and shape (the “fit”) of the odorant originating from the work of Moncrieff [16, 18] and then further solidified by Amoore [16, 19, 20]. The concept is generally referred to as the shape or “lock and key” theory (in addition to various permutations).

An alternate theory that is nonetheless prominent, and somewhat controversial (e.g., [12]), is the vibrational theory whereby odorant recognition is considered to be based on the vibrational modes of the molecules. Vibrational theory has a long history, first formulated by Dyson [16, 21] in the 1930s and then updated in the 1960s and later by Wright [16, 22–24]. It eventually fell out of favor due to lack of support [16, 25] but has been revived of late by Turin [26] and given further impetus recently [7, 11, 14, 27] with some truly remarkable and astonishing results obtained from  *Drosophila melanogaster* subjects [11]. From its original inception, the vibrational theory has been variously modified, and these modifications include acceptance of some components of shape recognition [26] (along with the “swipe card” model [13, 14, 27]) and actuation (inelastic electron or phonon-assisted tunneling [14, 26, 27]) of the ORs.

One of the approaches by Turin [7, 11] in assessing the mode of odorant recognition was to examine deuterated odorants. Though ^2^D (deuterium) isotopomers are highly interesting, they are not necessarily good tests for substantiating or invalidating the vibrational theory (vide infra). Herein, consideration is given to whether ^13^C isotopomers of odorants previously examined [11] can instead provide a decisive distinction between the competing shape and vibrational theories. This is based on the premise that ^13^C isotopomers do not induce significant changes to the IR frequencies—which has been the basis for arguments in favor of the vibrational theory using ^2^D isotopomers [7, 11]—but can nonetheless affect changes to other physical parameters such as Gibbs' free energies (Δ*G*s) or association/dissociation (on/off) rates of the binding of an odorant to a receptor (odorant-OR binding). The approach taken here was to model various ^2^D and ^13^C isotopomers as well as the ^1^H/^12^C (normal) isotopomers using DFT quantum chemical calculations within the framework of the *Gaussian09* program to predict their IR spectra as well as calculate their Δ*G* values. Modeling has been utilized previously [9, 11, 13, 14, 17, 26, 27] together with predicted IR spectra [9, 11, 14, 17, 26] for such purposes, but the changes to Δ*G* have generally been ignored or only the possibility of this being a significant factor quickly mentioned [17].

## 2. Background

As indicated above, the physiological aspects [2–4, 28, 29] with regards to odor detection have been established, and it has been determined that, for any particular organism, multiple ORs are present [2–5, 29] with the number of ORs varying greatly amongst species; for example, in human beings, it numbers about 350, and in the mouse there are about 1,000 ORs [4, 15]. Significantly, each OR can accept multiple odorants, and furthermore each odorant can activate multiple ORs [2–6, 29]. The result is that even with a relatively limited number of ORs, an almost unlimited number of potential responses are conceivable through a combinatorial system [2–6, 29]. Thus, the brilliant system that nature has come up with enables the detection of a vast number, and range, of odorants, including odorants never before encountered (i.e., never before produced in nature) which are nevertheless potentially detectable. Even for human beings which are considered to have a relatively weak sense of smell, a typical human being can discern tens, or even hundreds, of thousands of odors using only a few hundreds of ORs [4–6, 24]. As a consequence, the biological response in any particular organism is a highly complex one even before the consideration of experience and subjectivity (in human subjects at least), and other factors are taken into account. Thus, behavioral response studies of biological subjects are extremely challenging to make sense of because of the complexities involved [15], and such approaches are therefore unlikely to be definitive in identifying the mode of molecular recognition of the odorant, seemingly seminal results notwithstanding [11]. Hence, it is perhaps unsurprising as a consequence that despite decades of endeavor identification of the mode of odorant, recognition has yet to be accomplished unequivocally.

### 2.1. Enantiomer Differentiation

**Figure 1:**
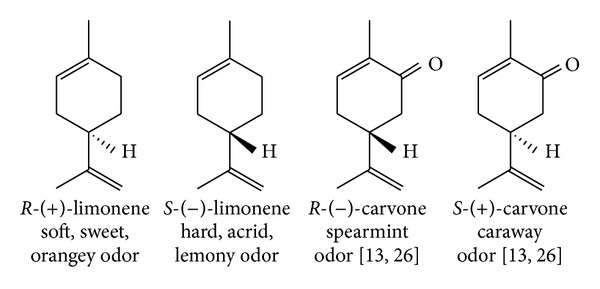
The structures of the enantiomer pairs of limonene and carvone. The evaluation of the odors of the limonene pair is the author's own perception.

Although well known and quite familiar to many people [13, 26] (classic examples are depicted in Figure 1), it has also been demonstrated that various other organisms can also discriminate, with varying degrees of capability with respect to various odorants, between two enantiomers (i.e., the two enantiomers of an enantiomeric pair in comparison to each other) with a number of examples being reported, for example, squirrel monkeys, rodents such as rats and mice, and fish [30].

Depending on how stringently the attributes of note and intensity are subjectively assessed, enantiomer pairs that smell the same can constitute anywhere between 5 and 59% of enantiomer pairs [13]. It is interesting therefore that the fact that some enantiomer pairs smell differently has been used as evidence against the vibrational theory [12, 17], whilst the fact that some smell the same has been used as an argument to substantiate vibrational theory [11, 26]. Ultimately, however, the question of olfactory discrimination of enantiomers is not particularly relevant and can be considered indeterminate. Since ORs, as biomolecules, are inherently chiral of course, therefore any model (and this has even been alluded to in modifications to the vibrational theory [26]) should give a differential response to some degree to chiral compounds, which may range from highly responsive to even indifferent. This is not dissimilar—and thus in line with observations—to enzymatic reactions which are often highly stereospecific or selective, but sometimes less so, and which can even exhibit variable stereospecificity [13]. Enzymatic reactions can, of course, even be relaxed with respect to substrate specificity.

### 2.2. Isotopomer Differentiation

**Figure 2:**
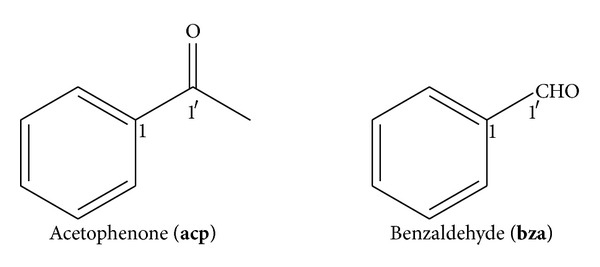
The structures of acetophenone (**acp**) and benzaldehyde (**bza**).

It has been demonstrated in a limited number of studies that some organisms can even discriminate, with varying degrees of capability, between various isotopomers of particular odorants. Reported examples include flies [11] and other insects [31], human beings [7, 9], and fish [32]. The most credible reports appear to be the recent reports with the insect *Drosophila melanogaster* [11] and human test subjects [7]. For the former subjects, the ability to distinguish isotopomers was ascertained by the use of electric shock training whilst for the latter, electric shock training was not required. In all studies, bar one, ^2^D was the abnormal isotope. In the one case where ^13^C was also used as the abnormal isotope [9], it should be noted that whilst comparisons of ^2^D, ^13^C, and ^1^H/^12^C (normal) isotopomers provided positive discernment for the ^2^D versus ^1^H/^12^C isotopomers, no discernment was observed in the ^13^C versus ^1^H/^12^C isotopomer comparison. However, the efficacy of the testing methodology used therein has been questioned [8, 17], and additionally further purification of the samples—to ensure chemical homogeneity as much as purity itself—was not undertaken after multiple sourcing of the isotopomers given that even trace impurities can produce anomalous results [13, 26]. The odorant tested in that study, benzaldehyde (**bza**, Figure 2), is not too distinct from acetophenone (**acp**), a molecule for which the ^2^D isotopomer was originally reported [26] to have been distinguishable from the ^1^H isotopomer by human test subjects and then determined not to be under rigorous testing [8] by other workers, following which the original claims were then retracted [7].

### 2.3. Vibrational Theory

**Figure 3:**
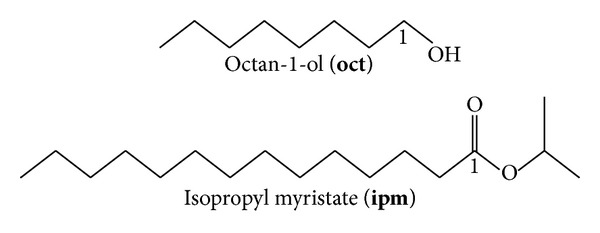
The structures of octan-1-ol (**oct**) and isopropyl myristate (**ipm**).

Vibrational theory asserts that odorants are recognized by the vibrational modes of the molecule. A mechanism by which this process occurs has been proposed [14, 26, 27], namely, inelastic electron or phonon-assisted tunneling. The basic tenet of the theory is that the vibrational modes of the molecule are able to take up the excess energy of an electron within the OR and enable it to translocate to a lower energy site; thus, ORs recognize, and are actuated by, odorants depending on the vibrational frequencies, they possess with the difference in energy between the two electron states being equal to the energy of the vibrational frequency. Antagonists to this theory highlight the fact that enantiomers should then all be perceived as having the same odor, whilst proponents argue that since most enantiomers do smell the same, this is consistent with the model. Since multiple ORs are involved, and the response to chirality can be muted (vide supra), the arguments, either way, are indeterminate. Opposing schools of thought have also both argued, in appropriate fashion, that vibrational spectra do [7, 11, 14, 22–24, 26]/do not [12, 16, 17, 25] correlate with predicted odors; similarly, opposing camps have both further argued, in appropriate fashion that molecular shape does [12, 17, 20, 33]/does not [6, 7, 11, 14, 15, 24, 26, 34] correlate with predicted odors. The concern of this work regards the question of isotopomers since the use of ^2^D isotopomers has been one of the cornerstones for substantiating the vibrational theory. This is because the replacement of ^1^H with ^2^D has a dramatic effect on the IR spectra, especially for bands involving C–H stretching since the reduced mass is altered by a factor of nearly 2 resulting in a reduction of the band frequencies by ca. a factor of $\sqrt{2}$. Thus, the seemingly spectacular results reported recently [7, 11] are truly astonishing and difficult to reconcile by any other theory. Not only were workers able to demonstrate conclusively that subjects (the fruit fly *Drosophila melanogaster* and human beings) could distinguish ^2^D isotopomers from ^1^H isotopomers (the former for the odorants acetophenone (**acp**) and octan-1-ol (**oct**); the latter for various musk odorants), but that with punishment training, the fruit flies could also transfer the aversion conditioning of one isotopomer (^1^H or ^2^D) to an analogous isotopomer of a different compound. The explanation for this was that the unique frequencies generated by the ^2^D isotopomer (in particular, the C–D bond stretch at ca. 2,220 cm^−1^) could be associated with an unpleasant experience, and thus taught to be avoided in another compound. Amazingly, the avoidance of these unique frequencies could even be transferred to compounds which have not been deuterated but which possess a functional group (nitrile in this instance) providing an IR band at the relevant frequencies. There are, however, concerns with the results and the subsequent interpretations. Whilst the ability to discern between ^1^H and ^2^D isotopomers is quite plausible, the transference to other compounds seems unlikely, especially when the flies are trained against ^1^H isotopomers since the medium for releasing the odorant, isopropyl myristate (**ipm**, Figure 3), also possesses C–H bonds in an extended chain similar to the test odorant **oct**. Workers have previously also pointed out the importance of the bond polarity in the inelastic electron tunneling process [26], yet the quite considerable differences in polarity between the C–D and C*≡*N bonds were not even considered in that study.

It is important to note that other factors besides a change in vibrational frequency can account for such results (vide infra), and therefore ^2^D isotopomers are perhaps not necessarily a final test for the validity of the vibrational theory, certainly not without extended examination.

### 2.4. Concentration Independency

**Figure 4:**
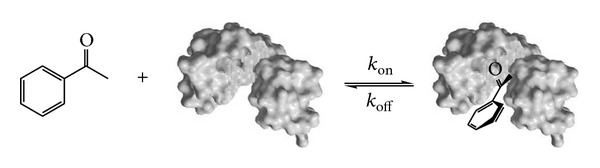
The binding of a ligand (**acp**) to a receptor (odorant-OR binding).

What is remarkable about the olfactory system is that although multiple ORs are used to sense an odor, the perception of that odor can remain essentially unchanged (with a minor number of notable exceptions) across a wide range of concentrations [29] despite, presumably, a set of different physical parameters in effect for the various ORs concerned which would then lead to varying responses from the ORs. Thus, there must be a regulatory system in place to handle these changes from the multiple ORs [29]. On the other hand, the observation that the perceived odor for some molecules is concentration dependent is attributed [4, 29] to the activation of additional receptors, thus implying the importance of binding affinity (and thus dependent on Δ*G*) and/or association/dissociation (on/off) rates (aside from habituation at high concentrations). These physical parameters are also wholly dependent on isotopomer identity as much as vibrational frequency and the observed changes could thus well have more to do with a shift in the equilibrium position of the odorant OR binding due to changes in Δ*G* or to the on/off rates for this process (Figure 4).

Hence, the key effect of isotopic substitution may be the disturbance of the regulatory system in terms of its performance for ^1^H isotopomers. Consequently, by way of changes to Δ*G* and/or on/off rates, the deuteration of very different compounds that have similar perceived odors may conceivably result in similar odor changes (e.g., musks [7]) that may have nothing to with vibrational frequency changes directly. Furthermore, the training of flies to prefer ^2^D isotopomers may then be directly transferable to other compounds given a choice of ^2^D or ^1^H isotopomers, as was demonstrated [11]. A more robust test might be to only train for one type of isotopomer rather than as pairs as was performed in that study in terms of this consideration. In the case of isotopomer training being transferable to other distinct compounds—citronellal and citronellyl nitrile—which happen to smell similar, it may just coincidentally be that the order of Δ*G* or the on/off rates are the same as for the isotopomers that the subjects were trained on.

Thus, an alternative approach to check for the dependency of odor on vibrational frequency could be by the use of ^13^C isotopomers since ^13^C isotopomers will not result in sizeable changes to the frequency of the associated IR bands as is the case for ^2^D isotopomers. However, it is difficult to attain sizeable Δ*G* changes without concomitant changes in IR frequencies. But since bonds are not broken in the detection process (i.e., the process of olfaction detection is a physical rather than a chemical process [16]), the same on/off rates can be expected for a ^2^D isotopomer as for a ^13^C isotopomer with the same number of isotopic substitutions since the changes in molecular weight are, for these purposes, the same. Since perceived odor is a combinatorial result of the actuation of a number of ORs, changing either of these aforementioned parameters can lead to a change in perceived odor. So, the question posed here is that can ^13^C isotopomers possibly settle the notion of a vibrational mode-based odor recognition process?

## 3. Results and Discussion

### 3.1. A More Generalized View of Binding

The “lock and key” description is potentially misleading as it may imply the need for a tight, or near perfect, fit. It is well known that enzymes are not always 100% stereospecific or selective and can even exhibit variable stereospecificity [35], an unsurprising result when enzymes can even be promiscuous with regards to the substrate, so a perfect fit is not necessary even for reactions. On the whole, there is unlikely to be a need for a tight fit, and generally while there might be high specificity required for some attributes, also at the same time there can be considerable tolerance for others by a particular OR [5, 15, 36]. Indeed, the odorant OR binding is known to be weak, at least in vertebrates [1, 26]. Furthermore, odor detection has also been shown to be temperature dependent [37], thus highlighting the weakness of the interaction and the dependency on either Δ*G* and/or on/off rates. This dependency also suggests against the vibrational theory unless molecular fit plays a significant role. Additionally, if high fidelity of fit was required, it would then be problematic on a functional level since it would then entail one specific OR per odorant; hence, an exhaustive number of ORs would then be required to sample the world at large. This is clearly not the case as tens, or even hundreds, of thousands of odors are perceived even by modest olfactory performers. And since it has already been demonstrated that for each odorant a number of ORs are used, a tight fit is hence inconceivable and the perfect fit concept is superfluous with regards to odorant binding. Bear in mind that it is generally accepted that there is direct physical contact between the odorant and the OR [1, 6, 15–17], and most theories now encompass some modem of shape and functional group dependency if they do not embrace it comprehensively, the implications are necessarily that there will be a shape component to the interaction, similarly a functional group dependency, and as the ORs are biomolecules, then as a consequence, chirality will also be contributing—strongly or weakly—to the shape recognition process. In other words, there is no reason to presuppose that odorant OR binding is substantially different to other usual ligand-protein-type binding interactions; that is, shape is necessarily a factor, but also the usual molecular interactions such as hydrogen bonding and van der Waals' attractive forces, as determined by the functional groups that are present in the odorant molecule [1, 5, 15]. Thus, varying degrees of latitude can be anticipated with regards to the fit which might be tight or loose, the dependency on topological polarization (i.e., the functional groups) which might be high or not, and thus the resulting binding affinity might be strong or relatively weak and easily moderated. These dependencies have been established by the examination of specific ORs, for example, rats [5] and humans [36]. In short, there are fit, functional group, and chiral components to the binding of an odorant. From this perspective, the profile-functional group (PFG) concept of Beets [38] is best equated to, or the description presented recently by Kaupp [1].

Thus, since an odor is perceived by an odorant activating multiple receptors, any change in the response of one of these receptors will result in an alteration to the perception of that odor. It is quite conceivable therefore that isotopic substitution may be disrupting the regulatory system that ensures that a perceived odor is invariant to concentration, either by changes to Δ*G* affecting the position of the equilibrium or molecular weight changes affecting the on/off rates. Since ^13^C isotopic substitution, unlike ^2^D isotopic substitution, has only minimal effect on vibrational frequencies and thus sizeable changes to Δ*G* are not realized, it could constitute a good test for observing any perceived odor changes due to on/off rates. For the vibrational theory to endure, it must at least be able to stand this test; though unfortunately if vibrational theory does indeed pass the test, it is not substantiated by it.

### 3.2. Modeling of the Odorants

**Figure 5:**
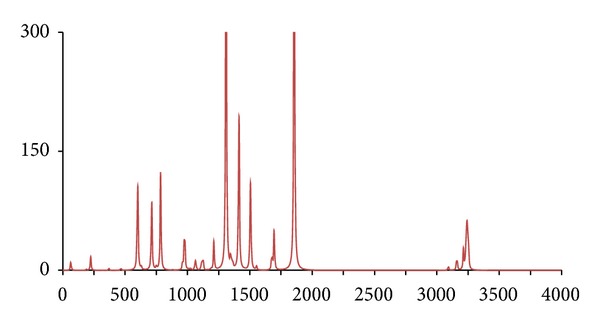
The DFT-calculated IR spectrum of acetophenone (**acp**). N.B. Bands at 1302 and 1848 cm^−1^ are offscale.

**Figure 6:**
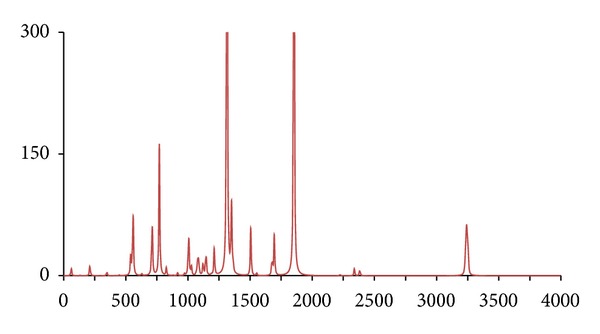
The DFT-calculated IR spectrum of *d*_3_-(H-2_3_′)-acetophenone (**acpd3**). N.B. Bands at 1316 and 1855 cm^−1^ are offscale.

**Figure 7:**
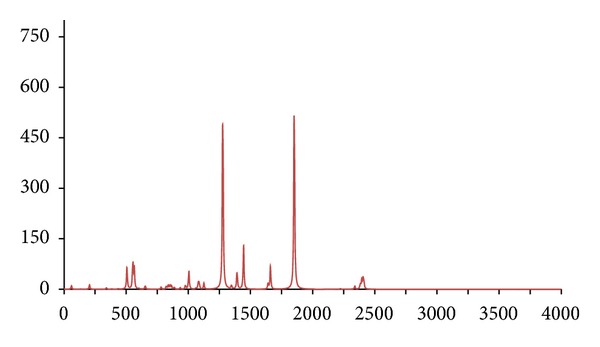
The DFT-calculated IR spectrum of *d*_8_-acetophenone (**acpd8**). N.B. Bands at 1275 and 1850 cm^−1^ are offscale.

**Figure 8:**
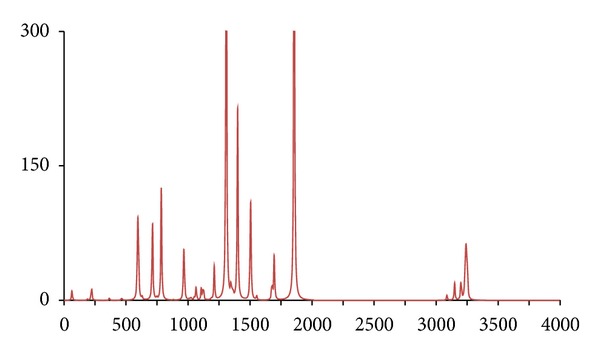
The DFT-calculated IR spectrum of (^13^C-2′)-acetophenone (**acpc1**). N.B. Bands at 1309 and 1855 cm^−1^ are offscale.

**Figure 9:**
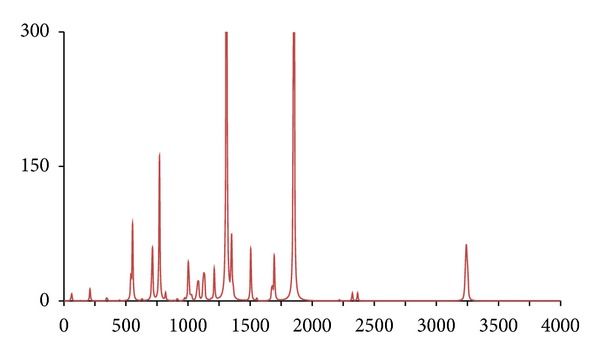
The DFT-calculated IR spectrum of *d*_3_-(H-2_3_′)-(^13^C-2′)-acetophenone (**acpc1d3**). N.B. Bands at 1309 and 1855 cm^−1^ are offscale.

**Figure 10:**
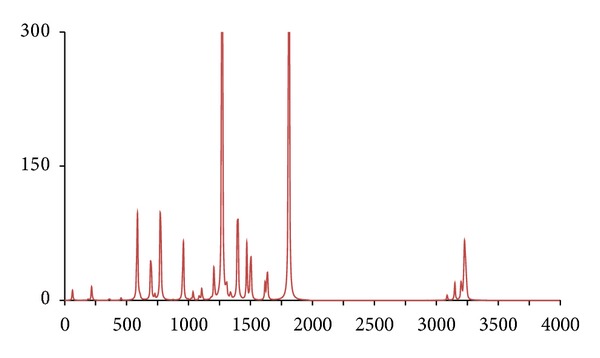
The DFT-calculated IR spectrum of (^13^C_8_)-acetophenone (**acpc8**). N.B. Bands at 1274 and 1806 cm^−1^ are offscale.

The molecular structures of acetophenone (**acp**) and octan-1-ol (**oct**) were modeled using DFT quantum chemical calculations within the framework of the *Gaussian09* program and their IR spectra predicted together with calculation of their Δ*G*s. For **acp**, in addition to the ^1^H/^12^C isotopomer, five other isotopomers, *d*_3_-(H-2_3_′) (**acpd3**), *d*_8_ (**acpd8**), (^13^C-2′) (**acpc1**), *d*_3_-(H-2_3_′)-(^13^C-2′) (**acpc1d3**), and (^13^C_8_) (**acpc8**) isotopomers, were also calculated. The calculated IR spectra for the six isotopomers are presented in Figures 5, 6, 7, 8, 9, and 10.

In accordance with expectations and previous work, only fairly minor and inconsequential changes to the ^1^H/^12^C isotopomer are seen in the spectra of below 2,000 cm^−1^ for the five abnormal isotopomer spectra; this is of special note for the isotopomers incorporating ^13^C nuclei (**acpc1**, **acpc1d3**, and **acpc8**). The most notable changes were to the aliphatic and aromatic C–H stretches, with appropriate shifts of the aliphatic bands from ca. 3,200 cm^−1^ to ca. 2,350 cm^−1^ for the **acpd3**, **acpd8**, and **acpc1d3** isotopomers and similar large shifts of the aromatic bands from ca. 3,250 cm^−1^ to ca. 2,400 cm^−1^ for **acpd8**. Most notable is the high similarity of the spectra for the **acp** and **acpc8** isotopomers, in line with the aforementioned assertions. These sizeable changes, and lack thereof, are mirrored in the calculated Δ*G*s (Table 1), only notable differences in Δ*G* for isotopomers involving ^2^D nuclei (**acpd3**, **acpd8**, and **acpc1d3**) are obtained relative to **acp**, even for **acpc8** with eight ^13^C nuclei. Obviously, then, changes in Δ*G* and IR frequencies essentially go hand in hand with respect to magnitude, and any change in Δ*G* can generally be considered inconsequential for ^13^C-only isotopomers. It is also worth noting that despite the very large change in Δ*G* for ^2^D isotopomers, only very slight, barely discernible differences (but statistically definitive nonetheless) have been reported by human test subjects [7], and similarly also for flies [11]. Thus, whilst significant changes would not be expected in terms of a shifting of the odorant OR binding equilibrium position for **acpc8**, it certainly seems that it would also have no effect on a vibration sensing system since such a system would have to be very sensitive to minor frequency changes, and the very opposite has in fact been postulated for the vibrational theory [11]. Since there are no bond breakages, changes in on/off rates for ^13^C isotopomers will be the same as for ^2^D isotopomers if the number of isotope substitutions is the same leading to the same nominal change in mass. Thus, changes in on/off rates would certainly be expected similar for **acpc8** as what would occur with **acpd8**. Thus, if this is the dependent parameter, both **acpd8** and **acpc8** would exhibit the same changes in biological response (or perceived odor if dealing with human test subjects) relative to **acp**. Moreover, since only minor changes are evident in the IR bands between **acpc8** and **acp**, this would then discount the changes in IR bands as the root cause and would hence discredit the vibrational theory.

**Figure 11:**
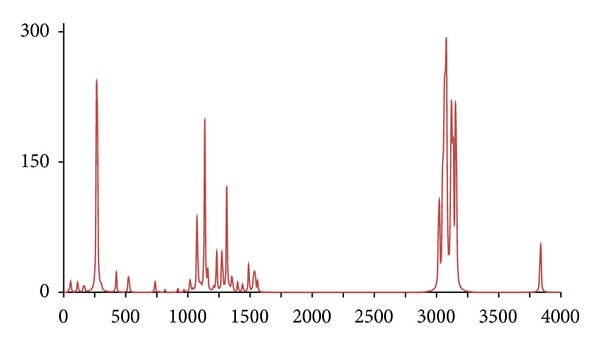
The DFT-calculated IR spectrum of octan-1-ol (**oct**).

**Figure 12:**
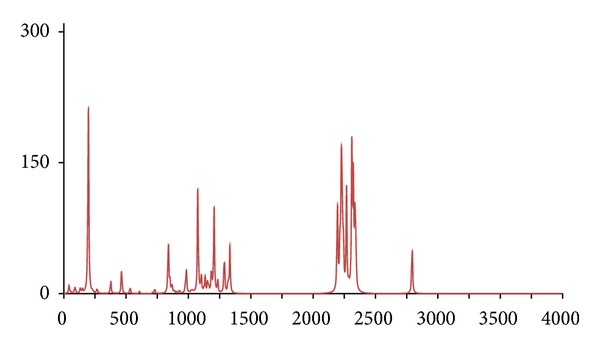
The DFT-calculated IR spectrum of *d*_18_-octan-1-ol (**octd18**).

**Figure 13:**
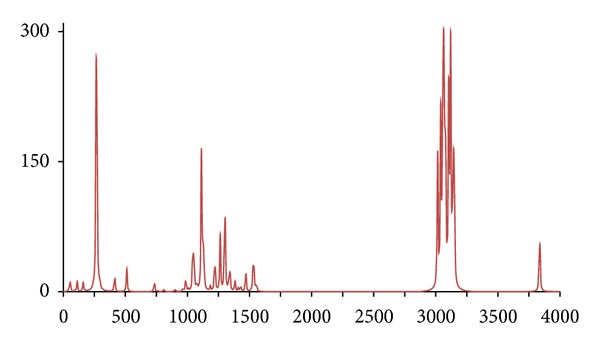
The DFT-calculated IR spectrum of (^13^C_8_)-octan-1-ol (**octc8**).

Similar calculations were conducted for the ^1^H/^12^C isotopomer **oct** and two isotopomers, *d*_18_ (**octd18**) and (^13^C_8_) (**octc8**), with similar results being obtained, namely, the IR spectrum of **octc8**  which was almost indistinguishable from **oct**, whilst for **octd18** large differences were only evident for the C–H and O–H stretches (Figures 11, 12, and 13).

**Figure 14:**
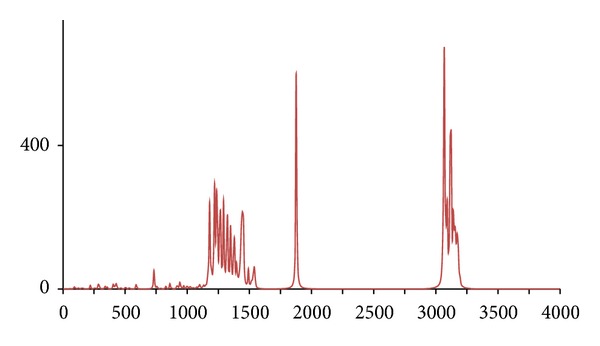
The DFT-calculated IR spectrum of isopropyl myristate (**ipm**).

**Figure 15:**
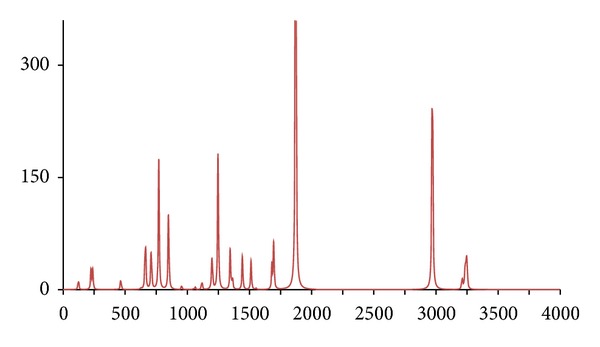
The DFT-calculated IR spectrum of benzaldehyde (**bza**). N.B. The band at 1869 cm^−1^ is offscale.

**Figure 16:**
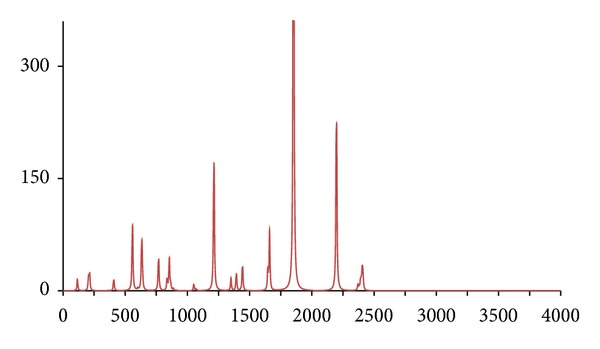
The DFT-calculated IR spectrum of *d*_6_-benzaldehyde (**bzad6**). N.B. The band at 1855 cm^−1^ is offscale.

**Figure 17:**
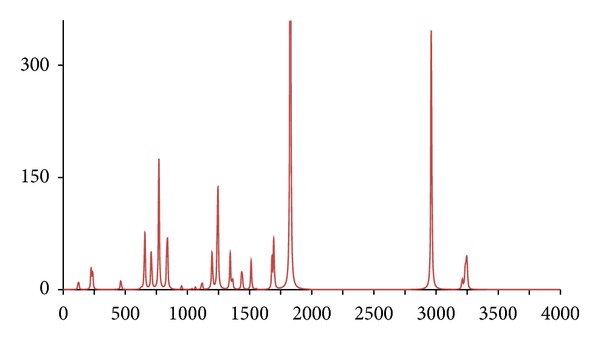
The DFT-calculated IR spectrum of (^13^C-1′)-benzaldehyde (**bzac1**). N.B. The band at 1820 cm^−1^ is offscale.

**Figure 18:**
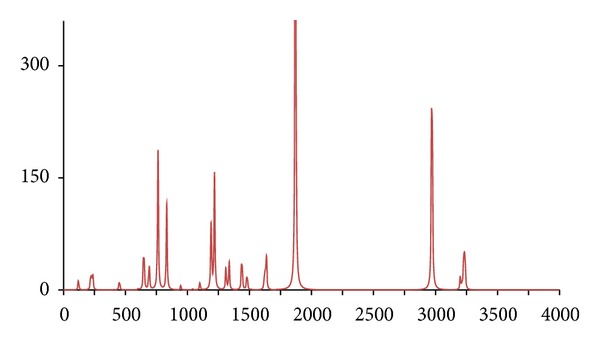
The DFT-calculated IR spectrum of (^13^C_6_)-benzaldehyde (**bzac6**). N.B. The band at 1869 cm^−1^ is offscale.

These variations were also appropriately mirrored by the calculated Δ*G*s (Table 1). Thus, similar conclusions can be drawn for **oct** as for **acp**. Incidentally, the medium used to convey the odorants in the *Drosophila melanogaster* study [11], isopropyl myristate (**ipm**), was also calculated to highlight the similarity of the C–H stretch bands (Figure 14)—in particular, to **oct**—and hence the seeming anomaly in that ^1^H isotopomers could be conditioned for aversion [11], if indeed these are the vibrations the flies are responding to. Interestingly, the change of Δ*G* per isotope nucleus is almost constant for both ^2^D and ^13^C nuclei (2 and 0.2 kcal mol^−1^, resp.) for the various isotopomers of both **acp** and **oct** (Table 1). Indeed, this pattern was continued with for benzaldehyde (**bza**). This compound was also modeled as it has been reported that the *d*_6_ isotopomer (**bzad6**) was distinguishable from the ^1^H/^12^C isotopomer (**bza**), whilst the (^13^C-1′) and (^13^C_6_) isotopomers (**bzac1** and **bzac6**) could not be [9]. It should be stressed, however, that there is doubt [8, 17] about these results, and experimental confirmation should be sought before drawing firm conclusions. Nevertheless, the IR spectra of these four isotopomers (Figures 15, 16, 17, and 18) displayed the expected differences and similarities, namely, ^13^C isotopomers **bzac1** and **bzac6** which were essentially indistinguishable from **bza**, and only for **bzad6** could sizeable and significant differences be observed for the aromatic and aldehyde C–H stretch bands. Likewise, only **bzad6** exhibited a sizeable difference to **bza** in terms of Δ*G* (Table 1). The results are very analogous to those obtained for **acp**, but whether the variation between ^1^H and ^2^D isotopomers constitutes a sufficient enough difference for human detection is open to conjecture at present.

## 4. Conclusions

The testing of isotopomers of various odorants has provided an intriguing set of results, but some aspects have seemingly been overlooked, namely, changes to the Gibbs' free energies (Δ*G*s) and association/dissociation (on/off) rates, both very important physical parameters for ligand-protein interactions. ^13^C isotopomers have the advantage over ^2^D isotopomers in that only minor changes in IR frequencies result, particularly for C–H stretching frequencies, but consequently then also only minor changes are attained for Δ*G*. However, similar biological response changes for the ^13^C and ^2^D isotopomers could be anticipated to arise from the on/off rates if they are the pertinent factor, and thus the testing of the ^13^C isotopomers of odorants that have exhibited differential responses between the ^1^H isotopomers and their deuterated analogues could potentially provide a pivotal test to effect a distinction between the competing shape and vibrational theories of odorant recognition. It is therefore proposed that the testing of ^13^C isotopomers can be conducted for such systems. For the vibrational theory to endure, it must pass this ^13^C isotopomer test. Unfortunately, though, whilst failure to do so would undermine the theory, passing it would not unequivocally validate the theory. Nevertheless, experimental verification is encouraged since the quintessential question that is yet to be comprehensively resolved is that which molecular property are the ORs responding to? This, despite broad acceptance, is indeed molecular fit (i.e., the shape theory).

## 5. Computational Procedures

DFT quantum chemical calculations were performed using *Gaussian09* [39] (version A.01) and analyzed using *GaussView* (version 4.1.2). The methodology and treatment of results followed that of Klika et al. [40]. Geometry optimization using tight convergence criteria, invoking the keywords *opt* = *tight* and *int* = *ultrafine*, of the structures in the gas phase was performed using the M06-2X hybrid metadensity functional [41, 42] with the 6–31G(d) basis set in tandem with vibrational analysis and thermochemistry calculations at the same level of theory. Vibrational analyses, invoking the keyword *freq* = *noraman*, to obtain the IR resonances also confirmed that optimized structures were true minima on the potential energy surface by not providing imaginary frequencies as well as providing the thermodynamic contributions at 298.15 K and 1 atm, wherein frequencies were left unscaled. IR spectra were generated by application of a broadening factor of 4 Hz and compiled for presentation using *Excel*.

## Figures and Tables

**Table 1:** Calculated Δ*G* values for various ^2^D, ^13^C, and ^1^H/^12^C isotopomers of acetophenone (**acp**), octan-1-ol (**oct**), and benzaldehyde (**bza**).

Compound and isotopomer	Δ*G*/kcal mol^−1^	Δ*G*/kcal mol^−1^ per D atom	Δ*G*/kcal mol^−1^ per ^13^C atom
Acetophenone (**acp**)	0.00	—	—
*d*_ 3_-(H-2_3_′)-Acetophenone (**acpd3**)	−6.41	−2.14	—
*d*_ 8_-Acetophenone (**acpd8**)	−17.18	−2.15	—
(^13^C-2′)-Acetophenone (**acpc1**)	−0.15	—	−0.15
*d*_ 3_-(H-2_3_′)-(^13^C-2′)-Acetophenone (**acpc1d3**)	−6.57	—	—
(^13^C_8_)-Acetophenone (**acpc8**)	−1.30	—	−0.16
Octan-1-ol (**oct**)	0.00	—	—
*d*_ 18_-Octan-1-ol (**octd18**)	−39.34	−2.19	—
(^13^C_8_)-Octan-1-ol (**octc8**)	−1.28	—	−0.16
Benzaldehyde (**bza**)	0.00	—	—
*d*_ 6_-Benzaldehyde (**bzad6**)	−12.82	−2.14	—
(^13^C-1′)-Benzaldehyde (**bzac1**)	−0.17	—	−0.17
(^13^C_6_)-Benzaldehyde (**bzac6**)	−0.98	—	−0.16
